# Extraction, Purification, Sulfated Modification, and Biological Activities of Dandelion Root Polysaccharides

**DOI:** 10.3390/foods13152393

**Published:** 2024-07-29

**Authors:** Xiao Wu, Na Li, Zeng Dong, Qin Yin, Tong Zhou, Lixiang Zhu, Hanxi Yan, Ziping Chen, Kefeng Zhai

**Affiliations:** 1 School of Biological and Food Engineering, Suzhou University, Suzhou 234000, China; szxywuxiao@163.com (X.W.); li60989211@126.com (N.L.); dongzeng@ahszu.edu.cn (Z.D.); qinyin@ahszu.edu.cn (Q.Y.); zhoutongy0117@163.com (T.Z.); zhulixiang0317@163.com (L.Z.); yanhanxi0217@163.com (H.Y.); 2 Engineering Research Center for Development and High Value Utilization of Genuine Medicinal Materials in North Anhui Province, Suzhou 234000, China; 3 Anhui Promotion Center for Technology Achievements Transfer, Anhui Academy of Science and Technology, Hefei 230031, China

**Keywords:** dandelion root polysaccharide (DRP), sulfation, structural characteristics, biological activity

## Abstract

In this study, polysaccharides were extracted at a rate of 87.5% ± 1.5% from native dandelion roots, and the dandelion root polysaccharides (DRPs) were then chemically modified to obtain sulfated polysaccharides (SDRPs) with a degree of substitution of 1.49 ± 0.07. The effects of modification conditions, physicochemical characterizations, structural characteristics, antioxidant properties, hypoglycemic activity, and proliferative effects on probiotics of DRP derivatives were further investigated. Results showed that the optimum conditions for sulfation of DRPs included esterification reagents (concentrated sulfuric acid: *n*-butanol) ratio of 3:1, a reaction temperature of 0 °C, a reaction time of 1.5 h, and the involvement of 0.154 g of ammonium sulfate. The DRPs and SDRPs were composed of six monosaccharides, including mannose, glucosamine, rhamnose, glucose, galactose, and arabinose. Based on infrared spectra, the peaks of the characteristic absorption bands of S=O and C-O-S appeared at 1263 cm^−1^ and 836 cm^−1^. Compared with DRPs, SDRPs had a significantly lower relative molecular mass and a three-stranded helical structure. NMR analysis showed that sulfated modification mainly occurred on the hydroxyl group at C6. SDRPs underwent a chemical shift to higher field strength, with their characteristic signal peaking in the region of 1.00–1.62 ppm. Scanning electron microscopy (SEM) analysis indicated that the surface morphology of SDRPs was significantly changed. The structure of SDRPs was finer and more fragmented than DRPs. Compared with DRPs, SDRPs showed better free radical scavenging ability, higher Fe^2+^chelating ability, and stronger inhibition of α-glucosidase and α-amylase. In addition, SDRPs had an excellent promotional effect on the growth of *Lactobacillus plantarum* 10665 and *Lactobacillus acidophilus*. Therefore, this study could provide a theoretical basis for the development and utilization of DRPs.

## 1. Introduction

Dandelion (*Taraxacum mongolicum* Hand.-Mazz.) is a kind of edible medicinal plant that has a reputation as a “natural antibiotic”, containing polysaccharides, terpenes, phenolic acids, flavonoids, coumarins, and other ingredients [1]. Among them, polysaccharides occupy an important position among nutrients, playing a crucial role in various biological activities within organisms. It has been shown that dandelion polysaccharides have antibacterial [2], anti-inflammatory [3], antioxidant [4], and antitumor [5] properties, as well as confer liver and gallbladder protection [6] and exhibit hypoglycemic effects [7].

In recent years, DRPs have been widely used in food and biomedical fields due to their multiple functions and biological activities, such as antioxidation [4], anticoagulation, blood sugar regulatory, immunomodulation, and antitumor [5,8] properties. Moreover, polysaccharides can be modified to generate new functional properties, changing their biological activities [1]. The modification methods mainly contain sulfate esterification [2], phosphorylation [3], acetylation [4], and carboxymethylation [5], which all bring about structural changes. A previous study reported that the garlic polysaccharides after phosphorylation had a more vital ability to scavenge hydroxyl radicals and superoxide anions than those without phosphorylation [6]. The acetylated cycloheximide polysaccharides significantly stimulated macrophage proliferation, possessing a much stronger ability than unmodified polysaccharides [7]. Utilizing sulfated groups linked to the long chain of green and red mushroom polysaccharides, sulfated green and red mushroom polysaccharides had good antioxidant, anticoagulant, antibacterial, and antitumor activities through in vitro activity experiments [8].

Sulfation was an easy and effective method to change the biological activity of polysaccharides by replacing their hydroxyl groups with sulfate groups [9]. It not only improved the water solubility of polysaccharides but also altered the conformation of the polysaccharide chain and bioactivities, especially the anticoagulant, antioxidant, antibacterial, and antitumor activities [9,10,11]. However, the research about modified DRPs is still at a primary stage, and few studies for sulfation of DRPs have been reported [12,13].

In this study, sulfated DRPs (SDRPs) were prepared from the dandelion root via the phenol-sulfuric acid method. Their effects were structurally characterized via Fourier transform infrared spectroscopy, high-performance liquid chromatography, and nuclear magnetic resonance spectroscopy. The antioxidant properties, hypoglycemic activity, and value-adding effect of SDRPs were also measured. The aim is to explore the new application fields of DRPs by studying the structure and bioactivity of SDRPs and provide theoretical references for an in-depth study of SDRPs and the development of dandelion resources.

## 2. Materials and Methods

### 2.1. Materials and Chemical Reagents

Dried dandelion roots were purchased from a Chinese herbal medicine shop (Suzhou, China); Trichloromethane, *n*-butanol, and anhydrous glucose were all purchased from Sinopharm Group Chemical Reagent Co., Ltd. (Wuhan, China). 1,1-Diphenyl-2-picryl-hydrazyl (DPPH) was purchased from Shanghai Maclin Biochemical Technology Co., Ltd. (Shanghai, China). Phenol, concentrated sulfuric acid, Kaumas Brilliant Blue G250, and anhydrous ethanol were all purchased from Beijing Solaibo Technology Co., Ltd. (Beijing, China). Activated charcoal, gelatin, barium chloride, and anhydrous potassium sulfate were all purchased from Shanghai Yuanye Biotechnology Co., Ltd. (Shanghai, China).

### 2.2. Extraction and Purification of DRP

#### 2.2.1. Sulfation of DRPs

DRPs were extracted via an ultrasound-assisted method using deionized water as the extractant, with a material-liquid ratio of 1:25 (m/V), ultrasonic power of 700 W, and ultrasonic time of 40 min. The extract was filtered, deproteinized, decolored, concentrated, ethanol precipitated, and lyophilized to obtain crude DRP [14].

The crude DRPs were purified with chromatography using DEAE-cellulose (16 mm × 500 mm) and glucose gel SephadexG-100 (16 mm × 500 mm) columns in sequence. Elution conditions for the two rounds of purification were as follows: (1) DEAE-cellulose column chromatography: the concentration of DRP was 20 mg/mL; the sample loading volume was 5 mL; five concentrations of elution were prepared at 0, 0.1, 0.2, 0.3, and 0.4 mol/L, respectively; elution flow rate was controlled at 1 mL/min; 6 mL was collected for each tube; and 10 tubes were eluted for each concentration [15]. (2) Glucose gel SephadexG-100 chromatography column: the sample loading volume was 5 mL; the sample was eluted with deionized water; the flow rate was controlled at 0.5 mL/min; and 3 mL was collected for each tube. The eluent was detected via the phenol-sulfuric acid method, polysaccharide elution peaks were combined, and finally, the extract was concentrated and lyophilized to obtain purified DRP [16].

(1) Sulfation reactions

A total of 2.5 mL of *n*-butanol was mixed with concentrated sulfuric acid at a certain ratio, put into a dry triangular flask, then added with 0.125 g of ammonium sulfate accompanied by sufficient stirring, and 0.5 g of dandelion polysaccharide was slowly added. The temperature of the system was maintained constant. The mixture was stirred for a certain period for adequate reactions, and its pH value was adjusted to 7–8 with 2.5 mol/L NaOH solution. Finally, it was dialyzed with a dialysis bag for 72 h, filtered, then further dialyzed with deionized water for 36 h, concentrated, and freeze-dried to obtain SDRPs [17].

The sulfur contents of the SDRPs were determined using the reported method [11]. A calibration curve was constructed with sodium sulfate as the standard. The degree of substitution (*DS*) was calculated using the equation below:

(1) $$DS=\frac{162\times {SO}_{4}^{2-}\%}{100-(\frac{96}{98}\times {SO}_{4}^{2-}\%)}$$

(2) Single-factor test

The effects of reaction time, reaction temperature, esterifier dosage, and amount of ammonium sulfate added on the degree of sulfate substitution in SDRPs were investigated separately [18].

(3) Experimental design for optimization

Based on the results of the single-factor experiment, three single factors (sulfation reaction time, ratio of esterifier dosage, and amount of sodium sulfate added) were investigated using Box–Behnken (BBD) RSM to find out the yield of SDRP [19]. These three factors were denoted by A, B, and C and divided into three levels, coded −1, 0, and +1. Design-Expert 13.0.1.0 Trial was used to perform data analysis and response surface plotting. The levels of test factors are shown in Table 1.

#### 2.2.2. Structure Determination of SDRP

##### FTIR Analysis

A total of 2 mg of DRP and 2 mg of SDRP were mixed with spectroscopic-grade potassium bromide powder, then ground and pressed into a thin slice for FTIR measurement. Infrared spectra in the range of 4000 to 500 cm^−1^ were obtained [20].

##### High-Performance Liquid Chromatography (HPLC)

The monosaccharide composition of DRP was analyzed using high-performance liquid chromatography (HPLC) [21,22]. Polysaccharide derivatization: 100 μL of polysaccharide at a concentration of 4–5 g/L was taken into a 5 mL tube, 100 μL of 4 mol/L TFA was added, and the tube was sealed with N_2_ and hydrolyzed in an oven at 110 °C for 2 h and then cooled. Next, 200 μL of methanol was added and blown dry with N_2_; this was repeated three times, the TFA was removed, and 50 μL of 0.3 mol/L NaOH solution was added to completely dissolve the residue. Finally, 50 μL of 0.5 mol/L methanol solution of PMP was added, vortexed, and mixed, and the reaction was carried out in an oven at 70 °C for 100 min.

Chromatographic conditions: The column was a ZORBAX Eclipse XDB-C18, 250 mm × 4.6 mm i.d., 5 μm, followed by a selection of 0.1 mol/L phosphate (pH 6.7) buffer–acetonitrile (83:17, *v*/*v*). Finally, the monosaccharide composition was determined at a column temperature of 30 °C, a detection wavelength of 250 nm, a flow rate of 1 mL /min, and an injection volume of 20 μL.

##### Molecular Weight Determination

The molecular weights of DRP and SDRP were measured using high-performance size exclusion chromatography (HPSEC). Put 50 mg of the sample in a 10 mL volumetric flask, dissolve it with the mobile phase, and set the volume. Chromatographic conditions included: column: Ultrahydrogel TMLinear 300 mm × 7.8 mm i.d.; mobile phase: 0.1 M sodium nitrate; flow rate: 0.5 mL/min; and column temperature: 40 °C [23,24].

##### Congo Red Test

A total of 5 mg of pure DRPs and SDRPs were dissolved in 2 mL of ultrapure water, and 2 mL of Congo red (80 μmol/L) solution was prepared with different volumes of 1 mol/L NaOH solution, namely 0, 0.1, 0.2, 0.3, 0.4, and 0.5. Maximum absorption wavelength was recorded in the wavelength range of 400–600 nm using a UV-Vis spectrophotometer [25,26].

##### NMR Analysis

DRPs and SDRPs were added to an NMR tube with D_2_O as the solvent and measured on a 600 MHz NMR spectrometer with a probe temperature of 60 °C. A total of 16 scans were performed, of which 0 were null scans. The pulse angle was 30°, and the relaxation delay time was 1.0 s. The ^1^ H and ^13^CNMR spectra of DRPs and SDRPs were recorded using a Bruker AV-400 NMR spectrometer (Bruker Instrumental Inc., Billerica, MA, USA) at 25 °C [27,28].

##### Scanning Electron Microscopy Analysis

A certain mass of DRP and SDRP dry powder was put on conductive gel on the slide, with scattered polysaccharide powder removed. After transferring the sample into the ion sputtering vacuum spray with a gold target for a period of time, we observed the sample under the electron microscope and adjusted the appropriate area in order to clearly observe the morphology of polysaccharides and generate pictures [29].

#### 2.2.3. Biological Activity of SDRP

##### Antioxidant Activity Assays

(1) 1,1-Diphenyl-2-picryl-hydrazyl (DPPH) radical scavenging activity assay

The DPPH radical scavenging activities of SDRPs were measured using a previously reported method with minor modifications [30,31]. In brief, 2.0 mL of samples at different concentrations (0.05–1.0 mg/mL) was added to 2.0 mL of methanolic DPPH solution (8 × 10^−5^ g/mL). Ascorbic acid (Vc) was used as the positive control. The mixture was adequately shaken and incubated at room temperature for 30 min in the dark. The absorbance was measured at 517 nm. The scavenging activity was calculated using the following equation:

(2) $$Scavenging\;activity\;(\%)=1-\frac{A_{1}-A_{2}}{A_{0}}\times 100\%$$

where *A*_0_ is the absorbance of DPPH solution in the absence of the sample, *A*_1_ is the absorbance of a mixture solution of the sample and DPPH, and *A*_2_ is the absorbance of the sample solution in the absence of DPPH. The experiment was repeated three times with duplicate samples.

(2) Superoxide anion radical scavenging activity assay

The superoxide anion radical scavenging activity of the polysaccharides was determined using the method described by Peng, et al., with a minor modification [32]. Briefly, 0.3 mL of polysaccharide solution at different concentrations (0.1, 0.5, 1.0, 1.5, and 2.0 mg/mL) was mixed with 3.9 mL of distilled water and 3.0 mL of 50 mM Tris-HCl buffer (pH 8.2). Then, the reaction mixture was incubated in a water bath at 25 °C, followed by the addition of 0.3 mL of 0.045 M pyrogallic acid to the solution. The change in absorbance (A/min) of the reaction solution was measured at 325 nm every 30 s for 5 min against a blank (distilled water and 50 mM Tris-HCl buffer instead of the sample). The following equation was used to calculate the superoxide radical scavenging activity:

(3) $$Scavenging\;activity\;(\%)=1-\frac{A_{1}}{A_{0}}\times 100\%$$

where *A*_0_ was the rate of change in the absorbance of the control group in the superoxide radical generation system, and *A*_1_ was the rate of change in the absorbance of the sample. The experiment was repeated three times with duplicate samples.

(3) Hydroxyl radical scavenging ability assay

The hydroxyl radical scavenging activities of the polysaccharides were determined using a reported method, with slight modification [33]. Briefly, 7.0 mL of samples at different concentrations (0.2, 0.4, 0.6, 0.8, 1.0, 1.2, 1.4, and 1.6 mg/mL) was mixed with 1.0 mL of 9.0 mM ferrous sulfate, 1.0 mL of salicylic acid–ethanol (9.0 mM) and 1.0 mL of 9.0 mM hydrogen peroxide. Then, the mixture was shaken and kept at 37 °C for 30 min in a dark room. The absorbance of the mixture was measured at 536 nm using a UV spectrophotometer. Vc was used as a positive control. Hydroxyl radical scavenging activity was calculated using the following formula:

(4) $$Scavenging\;activity\;(\%)=\frac{A_{2}-A_{1}}{A_{0}-A_{1}}\times 100\%$$

where *A*_1_ refers to ultra-pure water in place of the sample, *A*_2_ refers to the polysaccharide sample, and *A*_0_ is the ultra-pure water in place of the polysaccharide sample and H_2_O_2_.

(4) Metal chelating assay

The ferrous ion-chelating ability of DRPs and SDRPs was investigated with the method of Zhai et al. [34], with slight modifications. Samples with different concentrations (0.1–2.0 mg/mL) were mixed with FeCl_2_ (0.1 mL, 2 mM) and ferrozine (0.4 mL, 5 mM), shaken well, and kept still for 10 min at 25 °C. Then, the absorbance of the mixture was determined at 562 nm. EDTA-Na_2_ was used as a positive control. The Fe^2+^ chelating capacity was calculated with the following formula:

(5) $${Fe}^{2+}chelating\;ability=\frac{A_{0}-A}{A}\times 100\%$$

where *A*_0_ indicates the absorbance of the mixture solution without the sample, and *A* is the absorbance of the test sample mixed with the reaction solution.

##### In Vitro Hypoglycemic Activity

The α-glucosidase inhibitory activity and α-amylase inhibitory activity of DRP and SDRP were determined according to the methods in [35,36], and their inhibition rate was calculated.

(1) Inhibition of α-amylase activity

Using the DNS colorimetric method [35,36], the α-amylase inhibitory activity of DRPs and SDRPs was determined. The inhibition rate was calculated with the following formula:

(6) $$\;\alpha -amylase\;inhibitory\;activity\;(\%)=1-(\frac{A_{1}-A_{2}}{A_{0}-A_{2}})\times 100\%$$

where *A*_0_ was an equal volume of distilled water in place of the sample solution, *A*_1_ is an equal volume of phosphate buffer in place of the α-amylase solution, and *A*_2_ is an equal volume of distilled water and phosphate buffer in place of the sample solution and α-amylase solution, respectively.

(2) Inhibition of α-glucosidase activity

The α-glucosidase inhibitory activity of DRPs and SDRPs was measured [37]. The inhibition rate was calculated using the following formula:

(7) $$\alpha -glucosidase\;inhibitory\;activity\;(\%)=\frac{A_{0}-(A_{1}-A_{2})}{A_{0}}\times 100\%$$

where *A*_1_ represented the absorbance of the sample, *A*_0_ is the absorbance of the phosphate buffer solution in place of the polysaccharide solution, and *A*_2_ is the absorbance of the SDRP solution.

##### Studies on the Value-Added Effects of Probiotics

(1) Probiotic proliferation analysis

A total of 10 mL of MRS liquid medium without a carbon source was autoclaved. DRPs, SDRPs, and FOSs were dissolved in sterile water and added into MRS liquid medium after passing through a 0.22 μm microporous filter membrane so that the final mass concentration of sugar was 0, 5, 10, 15, and 20 mg/mL. The activated experimental strains were inoculated under an aseptic environment and cultured at 37 °C for 48 h. The optical density value of the culture medium at 600 nm was measured (OD_600_ nm). Fructo-oligosaccharide (FOS), a well-recognized prebiotic among multiple carbon sources which has a better proliferation-promoting effect on probiotics, was used as a positive control in this experiment [38,39].

(2) Probiotic growth curve

A total of 10 mL of MRS liquid medium without a carbon source was autoclaved. DRPs, SDRPs, and FOSs were dissolved in sterile water, and added into MRS liquid medium after passing through a 0.22 μm microporous filter membrane. Then, the final mass concentration of sugar was 20 mg/mL. *Lactobacillus plantarum* 10665 (LP10665) and *Lactobacillus acidophilus* (LA) were inoculated separately under aseptic conditions and incubated at a constant temperature of 37 °C for 48 h. Samples were taken at 6 h intervals, the pH value of the liquid medium and optical density value at the wavelength of 600 nm (OD_600_ nm) were measured, and growth curves were plotted [40].

## 3. Results and Discussion

### 3.1. Extraction of Crude DRP

The standard curve of DRP was plotted based on the absorbance value of a glucose standard solution at different concentrations [41], and the following regression equation was obtained: y = 1.421x − 0.0185, *R*^2^ = 0.9987. The absorbance of DRP solution was measured using the phenol-sulfuric acid method, and the extraction rate of DRP was 87.5% ± 1.5% according to the formula of the polysaccharide rate calculation. After deproteinization and decolorization of the DRP extract, the polysaccharide content was measured using the same method, and the DRP retention rate was 68.7% ± 2.0%.

### 3.2. Separation and Purification of DRP

**Figure 1:**
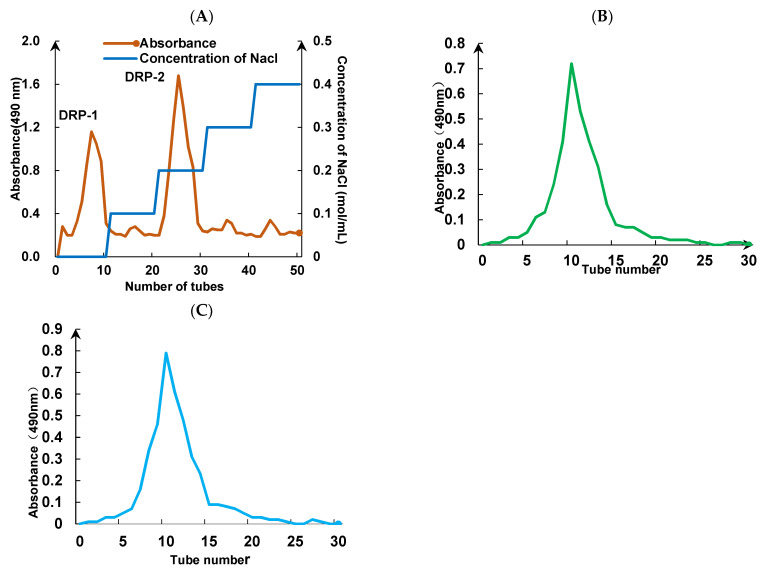
Separation (**A**) and purification (**B**,**C**) of dandelion polysaccharides.

Crude DRP was separated using a DEAE-52 cellulose column (Figure 1A). Fractions were obtained from eluted DRP in water, and the 0.1, 0.2, 0.3, and 0.4 mol/mL NaCl solutions had peak changes at 490 nm. The obvious absorbance peaks of two polysaccharides were named DRP-1 and DRP-2, with the two fractions accounting for 42.48% and 54.32%, respectively. DRP-1 and DRP-2 were then purified using a SephadexG-100 chromatographic column. Single elution peaks with good symmetry were obtained. Tubes with absorbance values greater than 0.1 were collected and freeze-dried to obtain the DRP fractions DRP-1 (Figure 1B) and DRP-2 (Figure 1C).

### 3.3. Sulfation of DRP and Optimisation of Conditions

#### 3.3.1. Single-Factor Test

**Figure 2:**
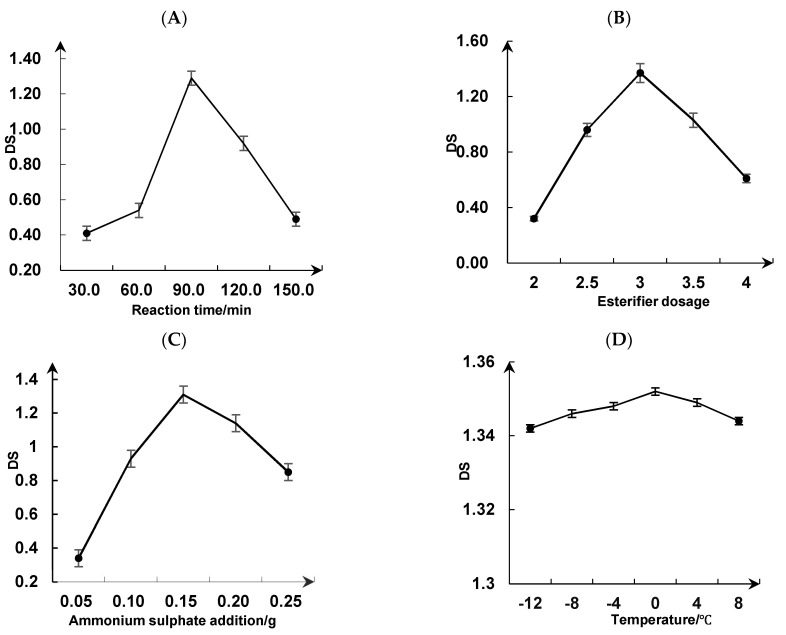
Effect of reaction time (**A**), esterifier dosage (**B**), ammonium sulfate addition (**C**), and reaction temperature (**D**) on the degree of substitution of dandelion polysaccharides.

SDRPs are derivatives of DRPs produced by replacing one or more hydroxyl groups on monosaccharide molecules in the macromolecular chain of DRP with sulfate [42]. As shown in Figure 2A, the degree of substitution increases and then decreases with the increase in sulfation reaction time. The degree of substitution had a maximum value of 1.29 when the reaction time was 90 min. Therefore, it was appropriate to choose 90 min as the reaction time for the sulfation of DRPs.

As shown in Figure 2B, the degree of substitution increases and then decreases with the increase in the ratio of esterifier addition. When the ratio of esterifier addition was three, the degree of substitution showed the maximum value at 1.37, and the degree of substitution decreased rapidly with the increase in the ratio of esterifier added. Therefore, it was more suitable to choose three as the ratio of esterifier added.

As shown in Figure 2C, the degree of substitution increases and then decreases with the addition of ammonium sulfate. When the amount of ammonium sulfate added is 0.15 g, the substitution degree appears to reach a maximum value at 1.31. The substitution degree changes more slowly when more ammonium sulfate is added. Therefore, the best amount of ammonium sulfate to add might be 0.15 g.

As shown in Figure 2D, the degree of substitution increases and then decreases with the increase in reaction temperature, but changes in the degree of substitution were small. When the reaction temperature was 0 °C, the substitution degree was 1.35, and the substitution degree decreased slowly with the increase in reaction temperature. Therefore, a reaction temperature of 0 °C was selected. According to the results in this figure, the influence of temperature on the degree of substitution was small. It was not used as a response surface optimization test factor.

#### 3.3.2. Response Surface Analysis

As shown in Table 2, experiments were conducted using Design-Expert 3.0.1.0 for response surface design. The entire design consisted of 17 randomly executed experimental points. By applying multiple regression analysis to experimental data, the response variable, and test variables were related by the following second-order polynomial equation, where Y was the degree of substitution of sulfate, and A, B, and C are reaction time, esterifier dosage, and the amount of ammonium sulfate added, respectively:

Y = 1.48 + 0.0025 × A − 0.0337 × B + 0.0413 × C − 0.2475×AB − 0.0375 × AC − 0.0200 × BC − 0.3695 × A^2^ − 0.2020 × B^2^ − 0.2770 × C^2^ (8)

F-test and *p*-value were used to measure the significance of coefficients in the model. In general, the model and variables in it were more significant at high absolute F-values and low *p*-values. As shown in Table 3, the model F-value was 132.95, and the *p*-value was <0.0001, which indicated that the model was highly significant. The lack-of-fit F-value and *p*-value were 1.73 and 0.2986, respectively, which indicated the model was suitable for predicting variations. In addition, the coefficients (B, C, AB, BC, A^2^, B^2^, and C^2^) were significant, with very low *p*-values (*p* < 0.05). This suggests that they had a significant effect on the average sulfate substitution of DRP. Based on the value of F, it can be deduced that the reaction time had the most significant effect on the degree of polysaccharide sulfate substitution, followed by the amount of ammonium sulfate added. Finally, the amount of esterifier added had the least significant effect, i.e., reaction time > amount of ammonium sulfate added > the amount of esterifier added.

**Figure 3:**
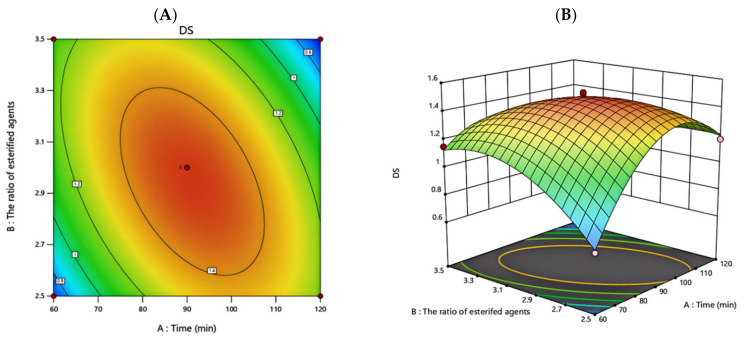

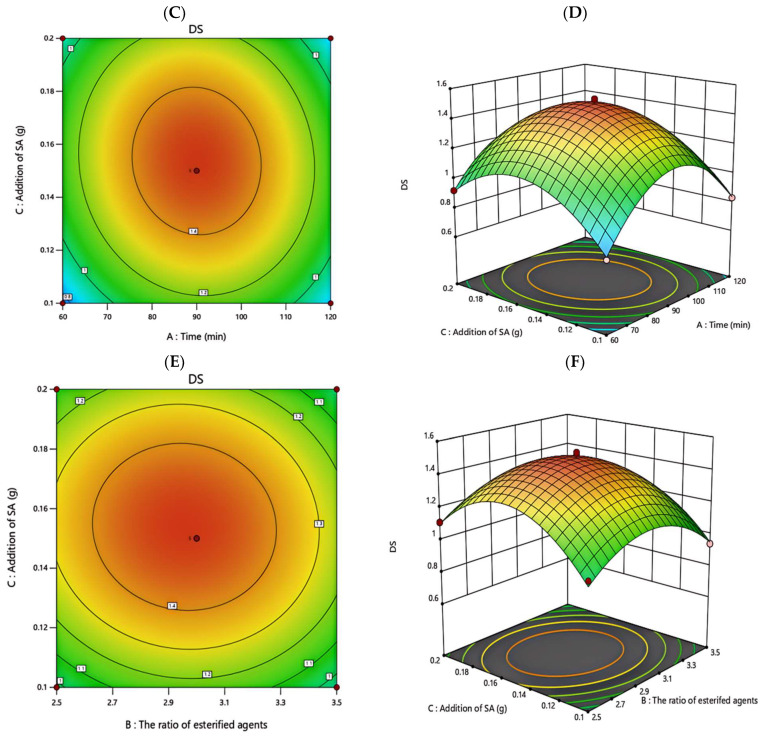
Contour plots (**A**,**C**,**E**) and three-dimensional response surface plots (**B**,**D**,**F**) about the effects of reaction time, esterifier dosage, the additional amount of ammonium sulfate, and interaction on the DS of sulfate of DRP.

3D response surface and contour plots were applied to predict the relationship between independent and dependent variables using Design-Expert (version 8.0), as shown in Figure 3. These plots provided a visual interpretation of interactions between two tested variables and the relationships between responses and experimental levels of each variable. The steep surface indicated the significance of the effects between variables. As shown in Figure 3A,B, the interaction of esterifier dosage ratio and reaction time on the degree of substitution was significant.

The experimental maximum was reached at an esterifier dosage ratio of three and a reaction time of 90 min. As shown in Figure 3C,D, the interaction effect of the amount of ammonium sulfate added and reaction time on the degree of substitution was apparent. The experimental maximum was reached when the amount of ammonium sulfate added was 0.15 g and the reaction time was 90 min. As shown in Figure 3E,F, the interactions of esterifier dosage ratio and the amount of ammonium sulfate added on the degree of substitution were more obvious. The experimental maximum was reached when the ratio of esterifier dosage was three and the amount of ammonium sulfate added was 0.15 g.

Based on the above analysis of the response surface, the following optimal conditions using the model equation were reaction time: 91.073 min; esterifier dosage ratio: 2.945; and amount of ammonium sulfate added: 0.154 g. Under optimal conditions, the degree of substitution was 1.487. For more validation, the predicted result was not biased towards the experimental value and a verification experiment was carried out under modified optimum conditions.

Experimental conditions for the actual operation were as follows: 0.5 g of DRP was added under the condition of an ice-water bath (0 °C). The reaction time was 91.073 min, with 2.5 mL of n-butanol, 7.363 mL of concentrated sulfuric acid, and 0.154 g of ammonium sulfate. Three parallel experiments were carried out, which showed the degree of substitution of 1.47 ± 0.005, with an error of 0.67%. This indicated that theoretical values fitted by the response regression equation had minimal deviation from actual values, proving that the process of esterification of DRP via the concentrated sulfuric acid method was operable.

### 3.4. Structural Characteristics

#### 3.4.1. FT-IR Analysis and Congo Red Test

**Figure 4:**
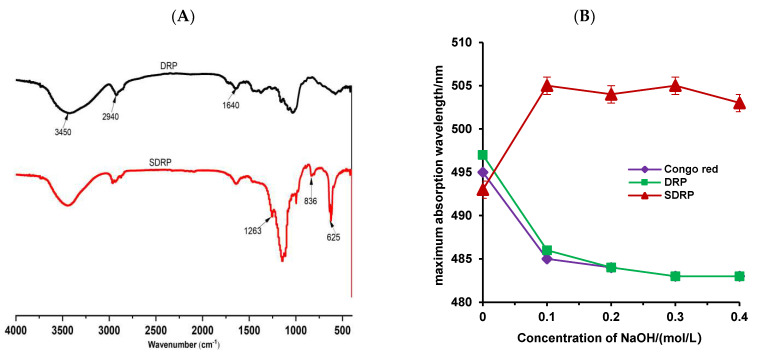
FT-IR spectra of DRP, SDRP (**A**), and Congo red test (**B**).

As shown in Figure 4A, DRP and SDRP absorption peaks were typical polysaccharide absorption peaks, and the main structure of DRP did not change before and after modification. The absorption peak at 3450 cm^−1^ is mainly caused by the stretching vibration of -OH in the polysaccharide molecule; the absorption peak at 2940 cm^−1^ is mainly caused by the stretching vibration of the C-H bond; and the absorption peak at 1640 cm^−1^ represents the stretching vibration of the carbonyl group (C=O) in the polysaccharide molecule. The difference is that, after sulfuric acid modification of DRP, there is a stretching vibration absorption peak of S=O bond at the vicinity of 1263 cm^−1^, a characteristic absorption peak of C-O-S stretching vibration at the vicinity of 836 cm^−1^, and a stretching vibration absorption peak of S-O bond at the vicinity of 625 cm^−1^, which was due to the conversion of the hydroxyl group to a sulfuric acid group. Therefore, these results indicated that sulfuric acid modification of DRP was successful.

As shown in Figure 4B, the ordered helical conformation of Congo red binds to polysaccharides to form complexes, and the redshift in the UV-visible maximum absorption wavelength determined the presence or absence of a three-stranded helical structure. There were no significant differences between DRP and Congo red control. Still, the maximum absorption wavelength of SDRP was highly increased at low NaOH concentration, suggesting a change in intermolecular forces with a triple-stranded helical structure. Therefore, it could be speculated that SDRP with *β*-1,3-glycosidic bonds can enhance the biological activity of polysaccharides when they form a triple helical conformation. These results can provide a structural basis for subsequent bioactivity studies.

#### 3.4.2. Monosaccharide Composition Analysis

**Figure 5:**
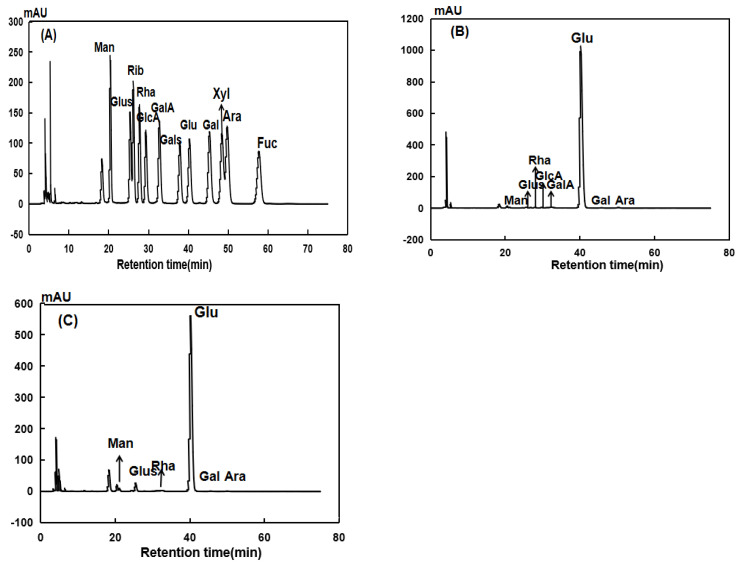
Chromatograms of monosaccharide compositions of standard substance mixture (**A**), DRP (**B**), and SDRP (**C**).

As shown in Figure 5, using high-performance liquid chromatography (HPLC) analysis, the retention times after derivatization with standard monosaccharides were compared to DRP, which was a type of heteropolysaccharide consisting of Man, Glus, Rha, GlcA, GlaA, Glu, Gal, and Ara, where the monosaccharides had a substance-to-volume ratio of 0.56:0.30:0.28:0.27:0.65:97.06:0.24:0.42. SDRP, also a heteropolysaccharide composed of Man, Glus, Rha, Glu, Gal, and Ara, had a similar monosaccharide composition as DRP. Still, the monosaccharides differ from each other in terms of ratio, at 1.82:2.98:94.55:0.26:0.25. The difference between DRP and their sulfated derivatives might be due to the degradation of the backbone and side chain in the sulfation process.

#### 3.4.3. Molecular Weight Analysis

The relative molecular mass (RMM) of polysaccharides was another important parameter influencing their bioactivities. The RMM of SDRP was measured using high-performance size exclusion chromatography (HPSEC). As shown in Table 4, the RMM of SDRP was lower than that of DRP, and the change in RMM provides some theoretical basis for the determination of the bioactivity of modified DRP.

#### 3.4.4. NMR Analysis

**Figure 6:**
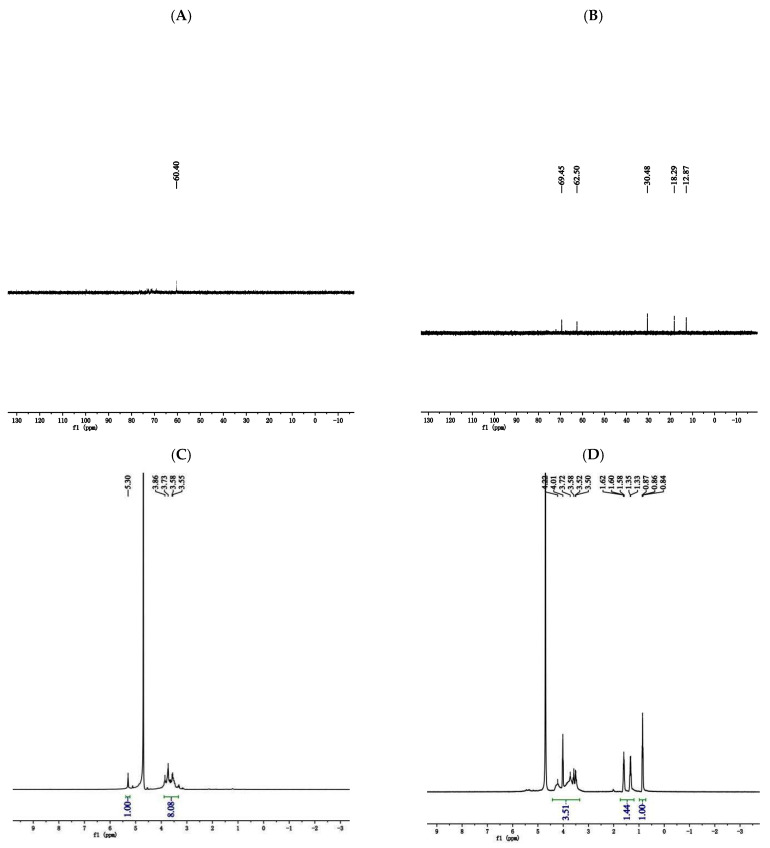
The NMR spectra of DRP and SDRP.((**A**,**B**) were the ^13^C NMR spectrum of DRPs and SDRPs respectively; (**C**,**D**) were the ^1^H NMR spectrum of DRPs and SDRPs respectively).

As shown in the ^13^C NMR spectrum (Figure 6A,B), compared with DRPs, SDRPs have signal peaks at 30.48 ppm, 18.29 ppm, and 12.87 ppm, which indicates that they underwent a chemical shift towards higher field strengths. This is consistent with the fact that carbon signals will shift towards higher field strengths when attached to electron-absorbing sulfuric acid groups. As shown in the ^1^H NMR spectrum (Figure 6C,D), chemical shifts between 4.3 and 5.9 ppm were the heterocapital hydrogen signals of polysaccharides, and tdense peaks in the region of 3.0–4.2 ppm were the characteristic peaks possessed by polysaccharides. These indicated that the overall structure of the polysaccharides has not been changed. Compared with DRPs, SDRPs underwent a chemical shift to higher field strengths. Characteristic signal peaks appeared in the region of 1.0–1.62 ppm, and chemical shifts in this region were C-S characteristic signal peaks, which were consistent with the results of 13C NMR spectra, further indicating that the sulfated modification was successful.

#### 3.4.5. Scanning Electron Microscopy Analysis

**Figure 7:**
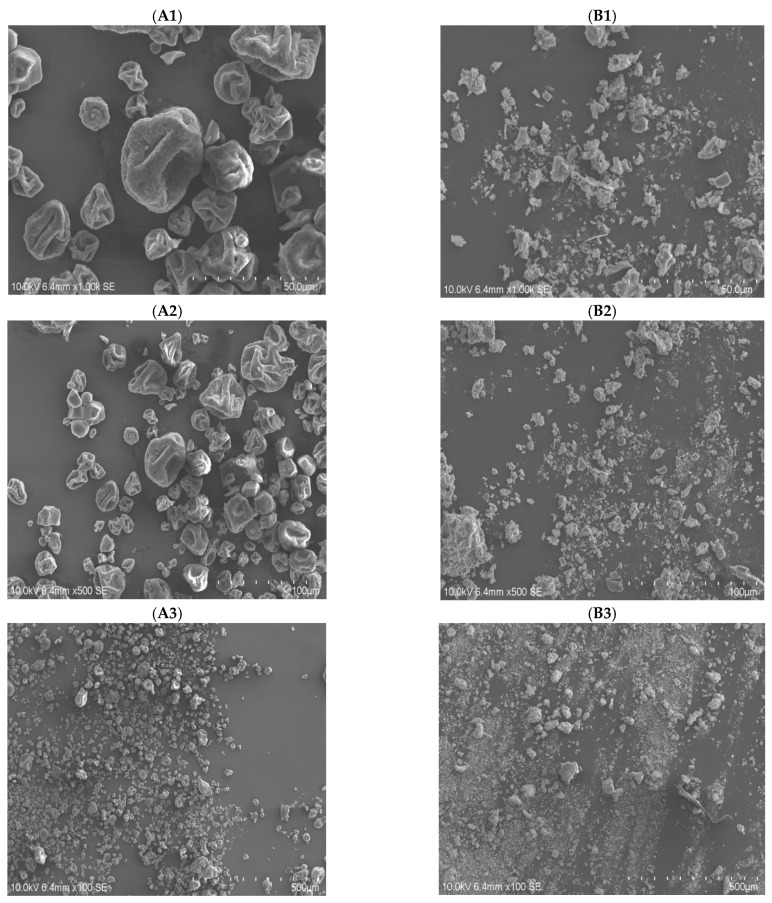
Scanning electron microscopy images of DRP (**A1**–**A3**) and SDRP (**B1**–**B3**).

As shown in Figure 7, the main morphology of DRP was a smooth and uniformly curled surface with homogeneous morphology, while the SEM image of SDRPs was in the form of fine fragments as compared with DRPs. This indicates that sulfated modification alters the spatial morphology of DRPs, contributing to their physiological activities.

### 3.5. Studies on the Biological Activity of SDRP

#### 3.5.1. Antioxidant Activity Analysis

**Figure 8:**
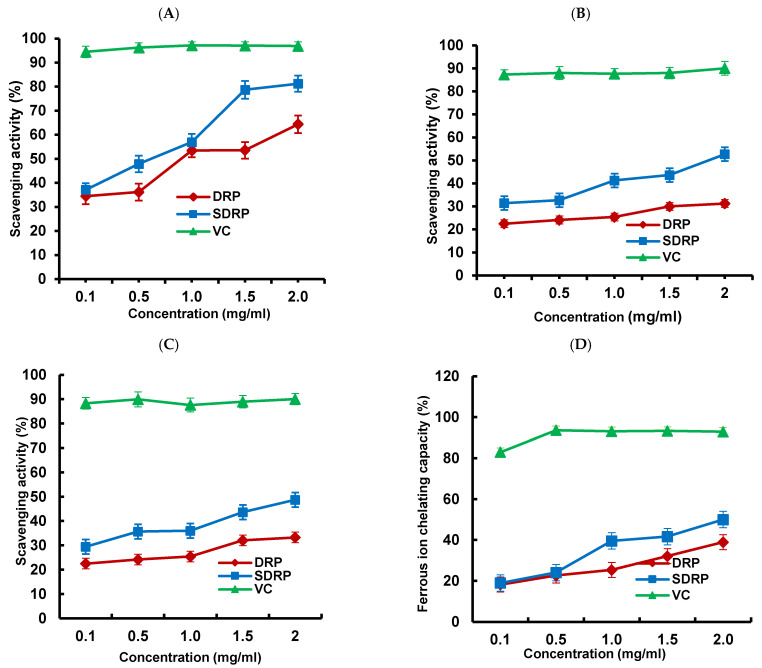
(**A**) DPPH radical scavenging assay; (**B**) superoxide anion scavenging activity assay; (**C**) hydroxyl radical scavenging ability assay; and (**D**) measurement of ferrous ion chelating capacity.

As shown in Figure 8A–C, the scavenging effect of DRP and SDRP on each radical was enhanced with increasing concentration. This may be for the following reasons: (1) polysaccharide molecules can enhance the activities of antioxidant enzymes; (2) the hydrogen of polysaccharide molecules combines with nearby free radicals to form stable free radicals, thus ending the free radical chain reaction, and thus, a potent free radical scavenging ability is realized [43]. As shown in Figure 8D, with increasing concentration, the chelating effect of both DRP and SDRP on Fe^2+^ increased. Finally, it tended to slow down. Because polysaccharide molecules bind essential metal ions such as Cu^2+^ and Fe^2+^ with free radicals, they reduced the number of free radicals. Therefore, it has a strong chelating capacity for Fe^2+^ [44].

Among the above four antioxidant tests, DRPs and SDRPs showed the best scavenging effect on DPPH radicals. The presence of glucuronic acid in polysaccharide molecules chelated metal ions. Subsequently, it scavenged DPPH radicals as analyzed in the literature. In contrast, the composition of the monosaccharides of DRPs and SDRPs contains more glucuronic acid, which may be the main reason for their strong scavenging ability for DPPH radicals [45]. In addition, it was observed that the scavenging ability of SDRPs for various free radicals was significantly higher than that of unsulfated DRP. The antioxidant activity of the polysaccharides was also related to their molecular weight. The more reducing hydroxyl groups in low-molecular-weight polysaccharides are able to react with free radicals. In contrast, high-molecular-weight polysaccharides have stronger intramolecular hydrogen bonding, which restricts the formation of hydroxyl groups, resulting in poorer antioxidant activity. As shown in Table 4, the RMM of SDRP was lower than that of DRP, which was inconsistent with the result that the antioxidant property of SDRP was higher than that of DRP [46]. These results suggested that sulfated modification can significantly improve antioxidant activities.

#### 3.5.2. Study of In Vitro Hypoglycemic Activity

**Figure 9:**
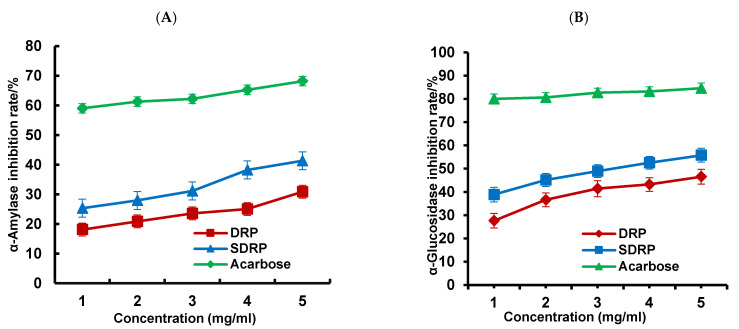
Studies on the hypoglycemic activity of sulfated dandelion polysaccharides in vitro ((**A**) Inhibition of α-amylase by polysaccharides and (**B**) inhibition of α-glucosidase by polysaccharides).

Inhibition of α-amylase activity slows the digestion of carbohydrates and reduces the rate of glucose uptake, thereby slowing the rise in blood glucose after a meal [47]. As shown in Figure 9A, the inhibition rates of α-amylase by DRPs and SDRPs were 30.96% and 41.77%, respectively, at a concentration of 5 mg/mL, and the inhibition rate of SDRPs increased by 10.81%. The reason may be that sulfation makes the molecular weight of dandelion polysaccharides smaller, which was more helpful for the release of active substances and higher hypoglycemic activity [48]. As shown in Figure 9B, as the concentration of DRPs and SDRPs increased, their α-glucosidase inhibition increased. At a concentration of 5 mg/mL, the inhibition rates of DRPs and SDRPs were 46.55% and 55.79%, and the inhibition rate of SDRPs increased by 9.24%. α-glucosidase plays an essential role in human glucose metabolism. Studies have shown that inhibiting their activity can effectively reduce the rate of carbohydrate absorption in the gut, resulting in a reduction in blood glucose levels [49]. Thus, it could be effective in lowering blood glucose by inhibiting the activities of α-glucosidase and α-amylase.

#### 3.5.3. Studies on the Value-Added Effects of Probiotics

**Figure 10:**
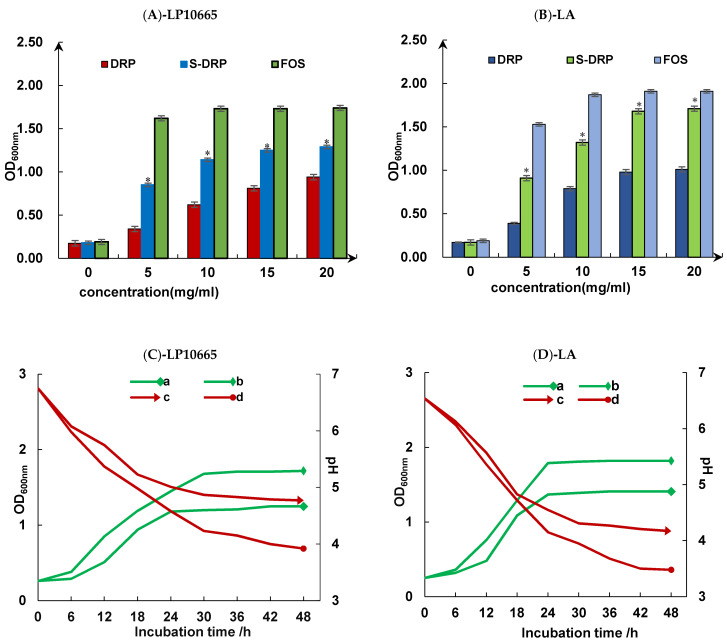
Probiotic proliferative effects (**A**,**B**) and probiotic growth curves (**C**,**D**). (**a**) SDRP medium OD_600nm_; (**b**) FOS mediumOD_600nm_; (**c**) SDRP medium pH; and (**d**) FOS medium pH. *: When the mass concentration of polysaccharides is the same, there is a significant difference (*p* < 0.05) between S-DRP, DRP, and FOS.

As shown in Figure 10A,B, when polysaccharide mass concentration varied in the range of 5-20 mg/mL, the numbers of *Lactobacillus acidophilus* and *Lactobacillus plantarum* 10665 increased with the increase in the mass concentration of SDRP after 48 h of constant temperature incubation at 37 °C. The growth trend of bacteria in the medium containing SDRP and FOS was the same, while the growth rate was greater than that in the medium containing DRP. This suggested that SDRP had a certain degree of proliferation promotion effect on both *Lactobacillus acidophilus* and *Lactobacillus plantarum* 10665. There was a positive correlation between the proliferation effect of probiotics and the mass concentration of SDRP within a certain range. When mass concentration reached 15 mg/mL, the rate of increase in the number of strains became slower. Then, the values and differences were compared and analyzed. In conclusion, the growth-promoting effect of SDRP on *Lactobacillus acidophilus* was better than that on *Lactobacillus plantarum* 10665.

As shown in Figure 10C,D, the OD_600nm_ values of SDRP medium and FOS liquid medium inoculated with *Lactobacillus plantarum* 10665 and *Lactobacillus acidophilus* both show a significant increase with incubation time. The OD_600nm_ values of both probiotics were basically stable after 36 h of incubation, and the overall trend of OD_600nm_ values was similar to that of the FOS-positive control group, which proved that the total number of probiotic colonies increased with the increase in incubation time within a certain range. When incubation time reaches 36 h, the growth rate of strain growth slows down. As can be seen from the graph line of the change in culture pH versus time, the pH values of *Lactobacillus acidophilus* and *Lactobacillus plantarum* 10665 in both the SDRP medium and FOS medium continued to decrease with the extension of incubation time, and the change in pH slowed down after the incubation time reached 36 h. The pH change values of both probiotics in the SDRP medium were closest to those of the positive control FOS group at 18 h of incubation. Then, the rate of pH decrease slowed down and gradually stabilized.

The results suggest that SDRP has a good pro-proliferative effect on *Lactobacillus plantarum* 10665 and *Lactobacillus acidophilus*, which can be considered as potential prebiotics.

## 4. Conclusions

In the present study, crude polysaccharides from dandelion roots were extracted via hot-water extraction, and isolated and purified to obtain the dandelion polysaccharides. Then, they were successfully sulfated using the concentrated sulfuric acid method. The series of biological activities were further explored. Results indicated that SDRP has a better promotion on antioxidant activity, hypoglycemic activity, and value-added effects of probiotics compared with DRP. These findings support the idea of the practical use of SDRPs for new application processing as a source of antioxidant and hypoglycemic agents in manufacturing in the pharmaceutical industry.

## Figures and Tables

**Table 1:** Box–Behnken design of test factor levels.

Factor	Encoding	Level		
		−1	0	1
Reaction time (min)	A	60	90	120
Ratio of esterifier dosage	B	2.5	3	3.5
Sodium sulfate addition (g)	C	1.0	1.5	2.0

**Table 2:** Design and results of Box-Behnken experiments.

Number	A (min)	B	C (g)	DS
1	60	2.5	0.15	0.68
2	120	2.5	0.15	1.17
3	60	3.5	0.15	1.15
4	120	3.5	0.15	0.65
5	60	3	0.1	0.74
6	120	3	0.1	0.83
7	60	3	0.2	0.92
8	120	3	0.2	0.86
9	90	2.5	0.1	1.01
10	90	3.5	0.1	0.94
11	90	2.5	0.2	1.11
12	90	3.5	0.2	0.96
13	90	3	0.15	1.46
14	90	3	0.15	1.51
15	90	3	0.15	1.45
16	90	3	0.15	1.48
17	90	3	0.15	1.52

**Table 3:** ANOVA for the quadratic response surface model.

Origin of Variance	Sum of Squares	Degrees of Freedom	Mean Square	F-Value	*p*-Value	Significant
Model	1.46	9	0.1623	132.95	<0.0001	**
A	0.0000	1	0.0000	0.0410	0.8454	
B	0.0091	1	0.0091	7.46	0.0292	*
C	0.0136	1	0.0136	11.15	0.0124	*
AB	0.2450	1	0.2450	200.72	<0.0001	**
AC	0.0056	1	5.6 × 10^−3^	4.61	0.0690	
BC	0.0016	1	1.6 × 10^−3^	1.31	0.2899	
A^2^	0.5749	1	0.5749	470.92	<0.0001	**
B^2^	0.1718	1	0.1718	140.74	<0.0001	**
C^2^	0.3231	1	0.3231	264.66	<0.0001	**
Residual	0.0085	7	1.2 × 10^−3^			
Lack of fit	0.0048	3	1.6 × 10^−3^	1.73	0.2986	
Pure error	0.0037	4	9 × 10^−4^			
Cor. total	1.47	16				

** indicates highly significant differences (*p* ≤ 0.01). * indicates a significant difference (*p* ≤ 0.05). And no * indicates that the difference is not significant (*p* > 0.05).

**Table 4:** HPSEC results for DRP and SDRP.

Sample	Peak Number	Retention Time/min	Relative Molecular Mass	Relative Peak Area (%)
DRP	1	18.967	40,644	23.57
	2	20.299	965	76.43
SDRP	1	18.950	13,673	14.46
	2	20.729	446	28.57

## Data Availability

The original contributions presented in this study are included in this article; further inquiries can be directed to the corresponding authors.
